# Nitrate of Silver

**Published:** 1854-07

**Authors:** Geo. Watt

**Affiliations:** Xenia, O.


### DENTAL REGISTER OF THE WEST.

VOL. VII. JULY, 1854. No. 4

### Original Communications.

*Nitrate of Silver.* By GEO. WATT, M.D., D.D.S., Xenia, O.

An article on nitrate of silver appeared in the last number of the Register, from the pen of Dr. Whitney, the object of which, we are told, was "to draw out discussion on the subject."

The Doctor states, in the outset, that there is "a popular prejudice in the profession, "against the use of nitrate of silver." This is to be regretted.

We fear, however, that he does not use the proper means to remove this prejudice. If we were reviewing his article we would complain of its indefiniteness more than of anything else.

This prejudice, he says, arises in part "from the known proportion of nitric acid used in its composition, or rather, in its preparation." Now, the Doctor knows that nitric acid is a *component* part of nitrate of silver, that it is as really present in its composition as in its preparation. This sentence is, at least, ambiguous.

Again, he says, "Nitric acid is partly decomposed by combination with any metallic oxyd." Now the Doctor should reflect that the acid unites directly with metallic oxyds, and that in contact with the metals themselves, *a part* of it is decomposed. He should then frame his language according to his reflections; for nitric acid is never "*partly* decomposed." With the same want of thought, he tells us that "in fusing the crystals, the acid is *nearly decomposed.* Again, he says, "In a very weak solution it, (the nitrate), preserves animal matter, and prevents polished steel from tarnishing." By this remark, as well as by those above, he would, it seems, have us believe that in nitrate of silver there is no very active chemical agent, and that it will have little or no chemical action on the teeth. Accordingly, he tells us that by many experiments, he perceives no chemical effect upon the body of the tooth, even when immersed in a saturated solution.

We will only now remark that these experiments, *out of the mouth,* teach but little in regard to what would take place *within it.* This will appear evident if we remember that circumstances modify affinity.

Nitrate of silver is composed of 54 parts of nitric acid and 116 oxyd of silver. When in contact with any substance for which the acid has a greater affinity than it has for oxyd of silver, the nitrate is decomposed, the acid combines with the new substance, and the oxyd is set free, producing the blackened appearance mentioned by Dr. W. This being the case, it is evident that to correct the prejudice complained of, it is better to meet the issue by defining the cases and conditions requiring the use of the article, enjoining the necessary precautions to prevent abuse, and by describing *clearly* its modus operandi, thus enabling all to use, understandingly, a remedy which the good of their patients often requires.

In describing the *methodus medendi* of the nitrate, the Doctor is equally unfortunate, and fails, either for the want of a knowledge of, or a want of attention to the chemical properties of the materials spoken of. As a specimen, "It acts decidedly, and in a twofold way—in destroying the animal fibers, that in their ramifications through the body of the teeth become exposed and inflamed, and then by closing the mouth of the cells with the silver, which, in parting with its corrosive power, unites with oxygen and forms an inert metallic oxyd." Now, in the name of science, has silver any corrosive power to part with? Or does he mean that the nitrate, in parting with its "corrosive power," unites with oxygen? Well, the "corrosive power" of nitrate of silver is *nitric acid*, and the salt does part with that, under the circumstances described. But the silver had united with oxygen, forming the "inert metallic oxyd," as the first step in the process for nitrate of silver. When the nitrate is decomposed the oxyd is found just where the acid *deserts* it. Accordingly, when the acid unites with the enamel, dentine, and "animal fibers, it is deposited on the tooth, giving it a black color.

Again, he says, "The tooth being porous absorbs more or less of the nitrate, which soon oxydizes. Now just think of a *nitrate* containing already six equivalents of oxygen, being oxydized.

Farther down, the Doctor speaks of heating a tooth, previously immersed in a solution of the nitrate, to a degree "sufficient to fuse the silver." Will a tooth bear a blowpipe heat of 1873 degrees? Or does he mean that we shall place it under a blowpipe, with a heat sufficient to *decompose the oxyd of silver?*

We have noticed only a few of the indefinite expressions contained in the article. We regret that so many have occurred, as they impair its utility, and destroy its influence.

In many conditions of the teeth and mouth we consider the nitrate an invaluable remedy, not because its acid is "nearly decomposed," but because it is there in all its strength; not because it is a *mild* remedy, but because its energy and promptness adapts to the urgent demands of these conditions.

To overcome professional prejudice against this useful remedy, we should bear in mind that in it we have a heroic remedial agent, that therefore it should not be used unless required, and that even then, discrimination should be used in the mode of applying it.

I am sorry the article was not read before the association. I would like to hear the "minds of the brethren" on the subject. As the object was to elicit discussion, I have felt at liberty to make these remarks, with all frankness. Hoping soon to enjoy a personal acquaintance with Dr. W., I will close. As I like *open*, manly discussion, I will not shrink behind a fictitious signature. You may place my name at the beginning or end of these remarks, as you think proper.

